# Exploring emerging concepts in the pathophysiology of PCOS: microbiome dysbiosis and immunological dysregulation

**DOI:** 10.1530/RAF-25-0187

**Published:** 2026-08-11

**Authors:** Parijot Kumar, Michael W O’Reilly, Karen McGowan, Conor Harrity

**Affiliations:** ^1^ National Maternity Hospital, Dublin, Ireland; ^2^ RCSI University of Medicine and Health Sciences, Dublin, Ireland; ^3^ Beaumont Hospital, Dublin, Ireland; ^4^ Rotunda Hospital, Dublin, Ireland

**Keywords:** PCOS, microbiome, immune dysfunction

## Abstract

**Abstract:**

Polycystic ovary syndrome (PCOS) is a complex yet common endocrine disorder, affecting 5–20% of reproductive-age women, characterised by a combination of hyperandrogenism, anovulation, and metabolic dysfunction. This combination of factors has traditionally been assumed to be the causative factors for the subfertility associated with PCOS, but significant microbial dysbiosis and alterations in immune function are identified in women with PCOS, suggesting a more complicated multifactorial impact on reproduction. Microbiome alterations vary between different PCOS subtypes, suggesting that hyperandrogenism and insulin resistance can impact microbial communities. The immunological implications of these microbiome changes are still poorly understood, along with their impact on reproductive outcomes, such as implantation, miscarriage, and assisted reproduction success rates. Although therapeutic interventions are chosen based on their endocrine and metabolic effects, many of these also target the microbiome and have immunomodulatory effects. Understanding these microbiome–host interactions and immunological factors provides new insights into the complex spectrum of PCOS pathophysiology and provides the potential for novel therapeutic approaches.

**Lay summary:**

Polycystic ovary syndrome (PCOS) is a common hormonal condition affecting up to one in five women of reproductive age. It can cause irregular periods, excess hair growth, acne, and difficulties with fertility. Recent research suggests that PCOS not only is a hormonal disorder but also involves changes in the body’s bacteria, known as the microbiome, and disturbances in the immune system. These changes may contribute to inflammation, insulin resistance, and hormone imbalance. Understanding how the microbiome and immune system interact in PCOS could lead to new ways to diagnose and treat the condition. Future therapies may focus on restoring a healthy microbiome to improve metabolic and reproductive health.

## Introduction

Polycystic ovary syndrome (PCOS) is perhaps the most prevalent endocrine disorder among women of reproductive age, having significant implications on both metabolic health and fertility (Zhou *et al.* 2024), and has a global prevalence estimated at 10–13% (Su *et al.* 2025). The condition encompasses a spectrum of reproductive, metabolic, and hormonal abnormalities that adversely impact fertility, metabolic health, and overall quality of life. Clinically, PCOS is characterised by menstrual irregularities, such as oligomenorrhoea or amenorrhoea; symptoms of hyperandrogenism, including acne, hirsutism, and alopecia; and polycystic ovarian morphology (PCOM) detectable on ultrasound examination (Su *et al.* 2025).

The pathogenesis of PCOS is complex and multifactorial, involving a combination of genetic predisposition, endocrine dysfunction, metabolic disturbances, and environmental influences. Disruption of the hypothalamic–pituitary–ovarian axis results in increased luteinising hormone (LH) secretion, enhanced ovarian androgen synthesis, and impaired follicular maturation (Su *et al.* 2025). Excess androgen production by ovarian theca cells gives rise to the clinical manifestations of hyperandrogenism, while insulin resistance – present in a substantial proportion of patients – further exacerbates androgen excess and contributes to an elevated risk of type 2 diabetes mellitus (Rosenfield & Ehrmann 2016, Singh *et al.* 2023). Chronic low-grade inflammation, increasingly recognised as a hallmark of PCOS, may amplify both metabolic and reproductive dysfunction (Singh *et al.* 2023, Su *et al.* 2025). These endocrine and inflammatory derangements are frequently accompanied by obesity, dyslipidaemia, and an increased risk of cardiovascular disease and endometrial carcinoma (Rosenfield & Ehrmann 2016, Su *et al.* 2025).

Diagnosis is most commonly established using the Rotterdam criteria, endorsed by the European Society of Human Reproduction and Embryology (ESHRE) and the American Society for Reproductive Medicine (ASRM). These criteria require the presence of at least two of the following features: oligo- or anovulation, clinical and/or biochemical hyperandrogenism, and PCOM – once other causes have been excluded (Smet & McLennan 2018, Christ & Cedars 2023, Teede *et al.* 2023). PCOM is defined as the presence of at least twelve follicles measuring 2–9 mm in diameter and/or an ovarian volume greater than 10 mL in at least one ovary. Recent guideline updates have incorporated anti-Müllerian hormone (AMH) levels as an alternative to ultrasound for defining PCOM (Teede *et al.* 2023). In adolescents, diagnosis requires the presence of both oligo-/anovulation and hyperandrogenism, as PCOM is often a physiological finding during puberty (Teede *et al.* 2023).

PCOS is a heterogeneous condition with several recognised phenotypes, classified according to the presence or absence of ovulatory dysfunction, hyperandrogenism, and PCOM (Christ & Cedars 2023). The clinical characteristics of these phenotypes vary, with differing presentations and variations in biochemical markers (Table 1). Phenotype A is most common in populations presenting to hospital settings (∼50%), while phenotype C is the most common in the general population (Mumusoglu & Yildiz 2020). The classic forms, phenotypes A and B, are generally associated with more severe reproductive and metabolic abnormalities, including marked insulin resistance, obesity, and metabolic syndrome. Normo-androgenic PCOS, corresponding to phenotype D, presents with ovulatory dysfunction and PCOM in the absence of androgen excess and tends to have a milder metabolic profile (Unfer *et al.* 2024). Additional subtypes, such as insulin-resistant, inflammatory, ‘post-pill’, adrenal, and lean PCOS, have also been proposed in the literature, reflecting further heterogeneity within the syndrome (ESHRE/ASRM 2004).

**Table 1:** Rotterdam phenotypes of polycystic ovary syndrome and associated clinical characteristics.

Phenotype	Hyperandrogenism	Ovulatory dysfunction	Polycystic ovarian morphology	Common description	Clinical characteristics
A	Yes	Yes	Yes	Classic/complete PCOS	Most severe phenotype; associated with higher rates of insulin resistance, obesity, and metabolic syndrome
B	Yes	Yes	No	Classic non-PCOM PCOS	Hyperandrogenic and anovulatory phenotype without ultrasound evidence of PCOM; significant metabolic burden
C	Yes	No	Yes	Ovulatory PCOS	Preserved ovulatory cycles with hyperandrogenism and PCOM; often milder reproductive phenotype but persistent endocrine dysfunction
D	No	Yes	Yes	Non-hyperandrogenic PCOS	Ovulatory dysfunction with PCOM in the absence of androgen excess; generally milder metabolic profile

This recognised heterogeneity has also prompted recent reconsideration of nomenclature. In 2026, an international global consensus process proposed renaming PCOS to polyendocrine metabolic ovarian syndrome, reflecting the broader endocrine, metabolic, and ovarian manifestations of the condition and addressing concerns that the term ‘polycystic ovary syndrome’ may not fully capture its multisystem nature (Teede *et al.* 2026). As much of the current literature, including the studies discussed in this review, continues to use the term PCOS, this terminology is retained throughout for consistency and clarity.

PCOS is one of the leading causes of anovulatory infertility worldwide. In addition to ovulatory dysfunction, metabolic derangements and systemic inflammation may impair oocyte quality and endometrial receptivity. Recent evidence has highlighted the potential role of the gut and reproductive tract microbiomes in the pathophysiology of PCOS. Alterations in microbial composition and diversity may influence systemic inflammation, insulin sensitivity, and steroid hormone metabolism, thereby affecting both metabolic health and reproductive potential. These observations have generated increasing interest in microbiome-targeted interventions as potential therapeutic strategies. The following section will explore these associations in detail, with particular emphasis on the interplay between microbial dysbiosis and fertility outcomes in women with PCOS.

### Microbiome and PCOS

Emerging evidence highlights that reproductive tract microbiome dysbiosis is prevalent in PCOS, and this may play a role in disease progression (Rizk & Thackray 2020, Gu *et al.* 2022). The human microbiome is a complex ecosystem, influencing host metabolism, immune function, and hormonal regulation (Rowland *et al.* 2018). In healthy women, the vaginal microbiome is typically dominated by *Lactobacillus* species, which maintain an acidic environment and provide protection against pathogenic organisms (Eslami *et al.* 2024). The gastrointestinal microbiome produces metabolites such as short-chain fatty acids (SCFAs) that regulate glucose metabolism, inflammation, and hormone synthesis (Zhou *et al.* 2024). Recent studies have demonstrated significant alterations in the human microbiome composition of PCOS patients compared with fertile controls, suggesting a potential bidirectional relationship between microbial dysbiosis and disease pathogenesis, with PCOS altering the microbiome, and the microbial environment influencing disease progression (Lu *et al.* 2021, Gu *et al.* 2022, Sun *et al.* 2023). Both compositional differences in species and functional alterations in microbial metabolism are demonstrated, potentially contributing to the characteristic features and symptoms of PCOS (Yang *et al.* 2021, Zhou *et al.* 2024). A reduced overall gastrointestinal microbiome diversity is a consistent finding with PCOS compared with unaffected controls (Rizk & Thackray 2020, Sun *et al.* 2023). The α-diversity, which measures species richness and evenness, is significantly decreased in PCOS (Rizk & Thackray 2020). This reduction in microbial diversity is associated with subsequent metabolic dysfunction, so it may potentially serve as a biomarker for disease severity (Sun *et al.* 2023). At the phylum level, PCOS patients demonstrate altered ratios of major bacterial groups, particularly changes in the *Firmicutes*-to-*Bacteroidetes* ratio (Rizk & Thackray 2020, Sun *et al.* 2023), with an increased abundance of *Bacteroides* species, which has also been demonstrated in animal models (Yang *et al.* 2021). Some beneficial bacteria, such as the S24-7 family, are negatively associated with PCOS (Rizk & Thackray 2020). Other beneficial bacteria, such as Butyricimonas, Blautia, Coprococcus, and Faecalibacterium prausnitzii, may all be significantly decreased. In contrast, an increase in potentially pathogenic organisms, such as *Escherichia–Shigella*, *Fusobacterium*, and the *Ruminococcus gnavus* group, has been reported (Zhou *et al.* 2024). These changes may have functional implications, with the depleted bacteria being primary producers of SCFAs with crucial roles in metabolic regulation and inflammation control (Kukaev *et al.* 2024, Zhou *et al.* 2024) (Table 1).

The reproductive tract microbiome encompasses distinct vaginal, cervical, and endometrial environments, all of which may be altered in patients with PCOS (Tu *et al.* 2020, Gu *et al.* 2022, Lee *et al.* 2025). The most consistent finding is a significant reduction in *Lactobacillus* species across all sites of the female reproductive tract (Tu *et al.* 2020, Lu *et al.* 2021, Gu *et al.* 2022). This appears particularly pronounced for *Lactobacillus crispatus*, whereas *Lactobacillus iners* may show variable changes depending on individual hormonal and metabolic status (Hong *et al.* 2021). In the vaginal microbiome, women with PCOS have demonstrated increased abundance of potentially pathogenic organisms, such as *Gardnerella vaginalis, Chlamydia trachomatis, Prevotella* species, and *Mycoplasma hominis,* which are commonly associated with vaginal dysbiosis (Tu *et al.* 2020, Gu *et al.* 2022). These alterations have been linked to adverse reproductive outcomes, including implantation failure, miscarriage, and preterm birth, although causal relationships remain uncertain (Gu *et al.* 2022, Eslami *et al.* 2024).

Emerging studies have also reported alterations in the uterine microbiome, with PCOS patients often exhibiting reduced endometrial *lactobacillus* dominance and increased diversity, which may create a less favourable environment for implantation (Ichiyama *et al.* 2021, Lee *et al.* 2025). However, these findings should be interpreted cautiously. The endometrial cavity represents a low-biomass environment, and sequencing studies remain susceptible to contamination from vaginal or cervical flora, reagent background signal, sampling variability, and inconsistencies in bioinformatic analysis. Consequently, the composition and clinical relevance of the endometrial microbiome remain areas of ongoing debate (Stoyancheva *et al.* 2025). Current evidence should therefore be considered preliminary rather than definitive. Interestingly, microbial alterations may also vary according to infertility phenotype. *Prevotella* was reported to be significantly reduced in PCOS patients compared with those with tubal factor infertility, suggesting an association with infertility (Zhao *et al.* 2025). PCOS patients with abnormal uterine bleeding may have predominantly anaerobic bacterial populations, which may be associated with an increased infection risk and compromised endometrial receptivity (Guo *et al.* 2025).

### Mechanisms for microbial dysbiosis

Hyperandrogenism plays a central role in modifying the microbiome of PCOS patients (Rizk & Thackray 2020, Yang *et al.* 2021, Zhou *et al.* 2024). An inverse relationship is seen between androgen levels and gastrointestinal microbial diversity, suggesting a potential negative impact of elevated testosterone on microbial structure (Rizk & Thackray 2020). This relationship appears to be independent of obesity or insulin resistance, suggesting specific hormonal effects (Rizk & Thackray 2020). Murine studies demonstrate that mice induced with PCOS have microbial changes affecting body mass and fat composition and that male-to-female faecal microbiota transplantation results in abnormal metabolic changes and elevated testosterone levels (Yang *et al.* 2021). Women with elevated testosterone have a higher abundance of *Lactobacillus crispatus* and lower levels of *Lactobacillus iners* in the vagina, possibly as an adaptive response to the altered hormonal environment (Hong *et al.* 2021, Lu *et al.* 2021). Nevertheless, it remains unclear whether hyperandrogenism drives these microbial alterations or whether microbial dysbiosis contributes to elevated androgen levels, suggesting a potentially bidirectional relationship that warrants further investigation (Chen *et al.* 2024).

A similar bidirectional relationship has been proposed for insulin resistance and hyperinsulinaemia. Microbial dysbiosis may impair intestinal barrier integrity and increase lipopolysaccharide translocation, promoting chronic low-grade inflammation and worsening insulin signalling, while insulin resistance itself may alter nutrient metabolism, bile acid homeostasis, and the intestinal microenvironment in ways that further influence microbial composition. Evidence from metabolic syndrome and insulin resistance outside the PCOS context suggests that some microbiome signatures may reflect broader metabolic dysfunction rather than being disease-specific (Qi *et al.* 2019). Similarly, sex-based microbiome differences and androgen-exposed animal models suggest that androgen signalling itself can shape microbial communities (He *et al.* 2021). Taken together, current evidence supports a potentially reciprocal relationship between endocrine dysfunction and microbiome alterations in PCOS rather than a simple unidirectional pathway, although longitudinal human studies are needed to clarify causality.

Insulin resistance is found in 50–70% of PCOS patients and has also been associated with gastrointestinal microbiome alterations (Yang *et al.* 2021, Mei *et al.* 2025). *Bacteroides* species are particularly linked to insulin resistance, with the potential for specific antibiotic treatment to reduce Bacteroides abundance, improving insulin sensitivity and glucose metabolism (Yang *et al.* 2021). This effect likely results from bacterial production of lipopolysaccharide (LPS) and disruption of intestinal barrier function (Parker *et al.* 2022, Zhou *et al.* 2024). The increase in gram-negative bacteria associated with PCOS can lead to excess LPS production, which then enters the systemic circulation, triggering chronic low-grade inflammation, due to a breach of the intestinal barrier (Duan *et al.* 2021, Zhou *et al.* 2024). This process activates Toll-like receptor 4 (TLR4) signalling pathways, which lead to an increased expression of pro-inflammatory cytokines, including TNF-α and IL-6 (Duan *et al.* 2021, Zhou *et al.* 2024). SCFA production is significantly reduced in PCOS patients with insulin resistance due to a decrease in Butyricimonas, Blautia, *Coprococcus*, and *Faecalibacterium prausnitzii*, which results in reduced levels of acetate, propionate, and butyrate, which are necessary for glucose regulation, insulin sensitivity, and maintenance of intestinal barrier integrity (Kukaev *et al.* 2024, Zhou *et al.* 2024).

### Microbiome differences within the PCOS spectrum

The phenotype of PCOS may be associated with changes in microbiome composition (Hong *et al.* 2021, Yang *et al.* 2024). The gastrointestinal microbiome composition in humans can vary significantly based on testosterone levels in PCOS patients (Yang *et al.* 2024). Patients with high-testosterone PCOS (PCOS-HT) have distinct microbial patterns compared with those with lower testosterone levels (PCOS-LT), suggesting a role of androgen status in shaping the microbiome (Yang *et al.* 2024). PCOS-HT patients have more abundant *Prevotella* and *Phocaeicola* species, while beneficial bacteria, such *as Alistipes, Agathobacter, Barnesiella, Faecalibacterium,* and *Ruminococcus,* are reduced (Yang *et al.* 2024). Conversely, PCOS-LT patients have elevated *Faecalibacterium, Bacteroides, Prevotella, Incertae Sedis,* and *Dialister* (Yang *et al.* 2024). These testosterone-associated differences are also associated with metabolic function, with PCOS-HT patients showing distinct correlations between bacterial composition and metabolic parameters (Yang *et al.* 2024).

Patients with specific clinical manifestations, such as acanthosis nigricans, abnormal uterine bleeding, and varying AMH levels, show distinct vaginal microbiome patterns (Hong *et al.* 2021). Significant regional variations are also reported in the microbiome composition of PCOS cases (Yang *et al.* 2024). In Chinese patients, *Gemmiger, Faecalibacterium, Burkholderia*, and *Duncaniella* show alterations compared with controls, with minimal changes in these specific bacteria in European populations (Yang *et al.* 2024). Additional data reported that *Alistipes* is more common in European patients, while elevated *Blautia* and *Roseburia* are seen in Chinese patients (Yang *et al.* 2024). With further research and understanding, there is potential that microbiome analysis could eventually be used to predict the clinical progression of the disease and response to treatment (Espinosa *et al.* 2024).

### Immune dysregulation in PCOS

**Figure 1:**
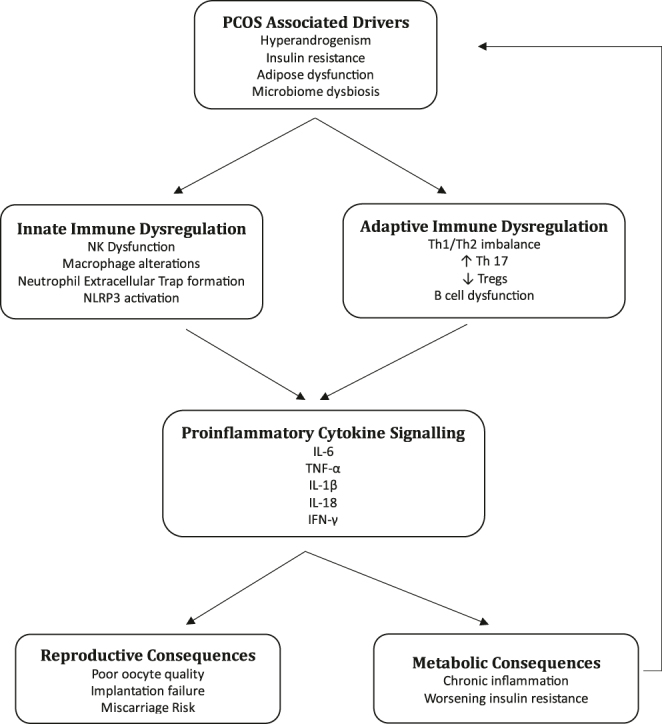
Proposed immune pathways contributing to reproductive and metabolic dysfunction in polycystic ovary syndrome.

PCOS is associated with a chronic low-grade inflammatory state, and although the associated microbiome alterations contribute significantly to this, other factors are also involved (Parker *et al.* 2022, Zhou *et al.* 2024). Significant immunological alterations and specific immune cell dysregulation are seen. The chronic inflammatory state associated with dysbiosis can lead to molecular mimicry and cross-reactivity between bacterial antigens and host tissues (Mousa *et al.* 2022). This may explain the increased prevalence of autoimmune conditions observed in women with PCOS (Mousa *et al.* 2022). Pro-inflammatory cytokines, including IL-6, IL-1β, IL-18, TNF-α, and IFN-γ, are elevated in both peripheral circulation and follicular fluid, contributing to a systemic inflammatory state (Banerjee *et al.* 2023, Wang *et al.* 2023). The inflammatory response also involves adaptive immunity, with PCOS patients showing increased numbers of T helper cells and altered T cell subsets (Sun *et al.* 2023, Guo *et al.* 2025). Other important findings include reduced regulatory T cells (Tregs), which suppress inflammation, increased pro-inflammatory T-helper 17 (Th17) cells, and abnormal B-cell activity with increased autoantibody production (Wang *et al.* 2023). The proposed interactions between endocrine dysfunction, immune dysregulation, inflammatory signalling pathways, and downstream reproductive and metabolic consequences in PCOS are summarised in Fig. 1.

Androgen excess drives tissue-specific immune changes: uterine natural killer (NK) cells increase, while adipose tissue NK cells show heightened activation markers (CD69) (Torstensson *et al.* 2024). There is a disrupted T cell balance with a skewed Th1:Th2 ratio (Wang *et al.* 2023), reduced uterine eosinophils (Torstensson *et al.* 2024), and altered NK cell activity (Torstensson *et al.* 2024). The local ovarian environment has a reduction in macrophages (Wang *et al.* 2023) and follicular fluid T cell deficiencies (CD4^+^/CD8^+^) (Wang *et al.* 2023). The NLRP3 inflammasome is activated in ovarian granulosa cells, driving IL-1β/IL-18 secretion through NF-κB pathway activation and causing mitochondrial dysfunction (Banerjee *et al.* 2023, Wang *et al.* 2023). Complement component is a central mediator of the immune dysregulation, with altered levels directly impacting inflammatory responses and cell viability (Zhang *et al.* 2024). Hyperandrogenism further modulates the immune changes by suppressing anti-inflammatory pathways while paradoxically promoting tissue-specific inflammation by altering adipose tissue immunity independent of obesity (Zhou *et al.* 2024) and other comorbidities (Banerjee *et al.* 2023, Wang *et al.* 2023). The chronic inflammation disrupts follicular development and endometrial receptivity, with Th1-dominant cytokine profiles in the follicular fluid impairing oocyte quality (Wang *et al.* 2023). Alterations to the uterine immunophenotype could potentially compromise implantation (Ye *et al.* 2022, Torstensson *et al.* 2024). A study found that within the endometrium of PCOS patients, the Th1 cell population are decreased, while Th2 cells are increased, with a possible impact on reproductive function (Guo *et al.* 2025).

Microbiome dysbiosis may also contribute to autoimmune phenomena as lipopolysaccharide from gram-negative bacteria activates the innate immune system through TLR4 signalling, leading to production of pro-inflammatory cytokines, including IL-6, TNF-α, and IL-1β (Duan *et al.* 2021, Zhou *et al.* 2024). These inflammatory mediators not only contribute to insulin resistance but also directly stimulate ovarian androgen production through upregulation of enzymes such as CYP17A1 (Zhou *et al.* 2024, Senthilkumar & Arumugam 2025). Neutrophil extracellular traps have been identified in the vaginal environment of PCOS patients with inflammatory changes (Espinosa *et al.* 2024). These structures, formed by activated neutrophils, can contribute to tissue damage and may impair fertility by creating a hostile environment for sperm survival and embryo implantation (Espinosa *et al.* 2024).

### Impact on reproductive outcomes

The combination of microbiome and immunological alteration associated with PCOS microbiome can influence fertility and the outcome of assisted reproduction (Ichiyama *et al.* 2021, Zhao *et al.* 2025). *Lactobacillus*-dominated endometrial environments are associated with better implantation rates, which may be a relevant factor for patients with reduced *Lactobacillus* abundance (Ichiyama *et al.* 2021). Specific bacterial species may be linked with poor outcomes, as *Lactobacillus* iners is negatively correlated with pregnancy rates, while some anaerobic bacteria may adversely impact embryonic survival (Zhao *et al.* 2025). Although endometrial analysis is becoming more widespread, vaginal *lactobacillus* rates may be more important biomarkers for implantation than endometrial levels (Ichiyama *et al.* 2021). PCOS patients yield more oocytes during IVF but exhibit lower fertilisation and cleavage rates due to inflammatory follicular environments (Ye *et al.* 2022). Endometrial immune dysregulation, such as elevated CD8^+^ T cells and dendritic cells, can correlate with poor embryo implantation rates (Ye *et al.* 2022, Wang *et al.* 2023).

PCOS patients with recurrent miscarriages may also have distinct microbiome patterns (Eslami *et al.* 2024). Increased levels of *Proteobacteria*, *Mobiluncus*, *Prevotella*, *Gardnerella vaginalis, Mycoplasma hominis*, and *Ureaplasma* have been associated with an increased risk of pregnancy loss, as has a decrease in *Lactobacillus* (Table 2) (Eslami *et al.* 2024). Immune factors are also important, as increased pro-inflammatory cytokines and reduced uterine eosinophils associated with hyperandrogenism may contribute to miscarriage risk by disrupting decidualisation and placental development (Torstensson *et al.* 2024). Recurrent pregnancy loss cases have significantly higher levels of pro-inflammatory cytokines, such as IL-2, IL-17A, IL-17F, TNF-α, and IFN-γ (Eslami *et al.* 2024). This inflammatory environment can disrupt the immune balance needed for the successful establishment and maintenance of pregnancy (Guo *et al.* 2025). Patients with hyperandrogenic phenotypes have higher risks, such as increased miscarriage rates along with a greater incidence of obstetric complications, such as hypertensive disorders, preterm birth, and fetal malformations (Guo *et al.* 2025). These adverse outcomes correlate with endometrial T cell dysfunction, suggesting a potential relationship, possibly associated with an altered microbiome–immune axis (Guo *et al.* 2025).

**Table 2:** Reported microbiome alterations in women with PCOS across gastrointestinal and reproductive tract sites.

Microbiome site	Microbial species increased in PCOS	Microbial species decreased in PCOS	Proposed implications	Strength of evidence
Gut	*Bacteroides* spp., *Escherichia–Shigella, Fusobacterium, and Ruminococcus gnavus* group	*Butyricimonas*, *Blautia*, *Coprococcus*, and *Faecalibacterium prausnitzii*	Reduced short-chain fatty acid production, increased lipopolysaccharide production, intestinal barrier dysfunction, insulin resistance, and chronic inflammation	Moderate
Vagina	*Gardnerella vaginalis, Prevotella* spp., *Mycoplasma hominis*, and *Chlamydia trachomatis*	*Lactobacillus* spp. (particularly *Lactobacillus crispatus*)	Vaginal dysbiosis, altered local inflammatory environment, and potential adverse reproductive outcomes	Moderate
Endometrium (emerging evidence)*	Increased diversity/anaerobes	*Lactobacillus*	Potential implantation failure and reduced endometrial receptivity	Limited

* Should be interpreted cautiously due to low-biomass sampling, contamination risk, and lack of standardised methodologies.

### Non-reproductive sequelae

Bile acid metabolism may also be impacted by PCOS-associated microbiome changes (Li *et al.* 2025). The intestinal microbiome deconjugates primary bile acids, converting them to secondary bile acids by bacterial enzymes (Li *et al.* 2025). *Bacteroides vulgatus* can be significantly upregulated in PCOS patients and is able to deconjugate conjugated bile acids, altering bile acid profiles (Li *et al.* 2025). The bile acid–microbiome axis may also influence the secretory function of intestinal type 3 innate lymphoid cells, regulating IL-22 secretion and intestinal immunity (Li *et al.* 2025).

PCOS patients have significantly reduced levels of acetate, propionate, and butyrate in both faecal and serum samples compared with controls (Kukaev *et al.* 2024, Zhou *et al.* 2024). These metabolites have important roles in glucose regulation, lipid metabolism, and control of inflammation. The reduction in SCFA levels is associated with a decrease in SCFA-producing bacteria, such as *Butyricimonas, Blautia, Coprococcus,* and *Faecalibacterium prausnitzii*, which have anti-inflammatory activity and maintain mucosal immune homeostasis (Kukaev *et al.* 2024, Zhou *et al.* 2024). Conversely, there is a documented report of overproduction of specific SCFAs in faecal samples of PCOS patients, particularly acetic acid and valeric acid (Kukaev *et al.* 2024). It was suggested that elevated levels certain faecal SCFAs correlated with obesity, while reduced serum levels could indicate impaired intestinal absorption or increased tissue utilisation (Kukaev *et al.* 2024).

The gastrointestinal microbiota can influence tryptophan metabolism and kynurenine pathway activity, with microbiome alterations in PCOS patients, potentially impacting tryptophan degradation and availability, contributing to chronic inflammation (Jovanovic *et al.* 2022). The activation of indoleamine 2,3-dioxygenase, which catalyses the first step of tryptophan degradation in the kynurenine pathway, is influenced by inflammatory signals and bacterial components (Jovanovic *et al.* 2022).

### Strength of evidence and limitations of microbiome studies in PCOS

Although emerging evidence increasingly supports an association between microbiome dysbiosis and PCOS, the current literature remains limited by important methodological and clinical heterogeneity. Most human studies are cross-sectional, which restricts the ability to infer temporal or causal relationships between microbial alterations and the endocrine or metabolic features of PCOS. Whether microbiome dysbiosis contributes directly to disease pathogenesis, develops secondary to hormonal and metabolic disturbances, or reflects a bidirectional interaction remains uncertain.

Interpretation is further made difficult by the heterogeneous clinical spectrum of PCOS. Distinct phenotypes defined by the Rotterdam criteria differ in androgen levels, ovulatory function, metabolic profile, and body composition, all of which may independently influence microbial composition (Zhu *et al.* 2025). Additional confounding factors – including body mass index, diet, ethnicity, medication exposure (particularly metformin or GLP-1 receptor agonists), and recent antibiotic or probiotic use – vary substantially between studies and may contribute to inconsistent findings.

### Therapeutic interventions

Several currently used PCOS therapies can indirectly or directly target the microbiome and immune dysfunction. Metformin is a commonly prescribed medication for these patients and has been demonstrated to have a significant impact on the gastrointestinal microbiome (Kukaev *et al.* 2024, Xu *et al.* 2025). Metformin can activate intestinal AMP-activated protein kinase (AMPK), leading to a positive modulation of the microbiome composition and an improvement in symptoms (Xu *et al.* 2025). Metformin can improve microbiome diversity, increase beneficial bacteria, and reduce potentially pathogenic organisms, helping optimise insulin sensitivity (Kukaev *et al.* 2024, Xu *et al.* 2025). The use of metformin for six months demonstrated restoration of beneficial bacteria, such as *Clostridium leptum* and *Prevotella*, while reducing opportunistic microorganisms, such as *Clostridium perfringens* and *Staphylococcus* species (Kukaev *et al.* 2024). Metformin also has immune-modifying properties, reducing TNF-α and IL-6 levels. The beneficial mechanism of metformin appears to involve activation of intestinal AMPK, which influences goblet cell secretion of antimicrobial peptides, affecting microbial homeostasis (Xu *et al.* 2025). This increases the production of beneficial metabolites, such as indole-3-carboxaldehyde (I3A), which has anti-inflammatory and anti-apoptotic properties that may benefit ovarian function (Xu *et al.* 2025).

GLP-1 receptor agonists, such as liraglutide or semaglutide, are increasingly used for weight management in PCOS cases and demonstrate microbiome-modulating effects in preclinical animal models (Sánchez-Garrido *et al.* 2024, Xiong *et al.* 2024, Voros *et al.* 2026). These studies reported changes in microbial composition, including marked decreases in both ACE and Shannon diversity indexes, occurring alongside improvements in insulin resistance, body weight, and androgen excess (Xiong *et al.* 2024). Importantly, these findings should be interpreted cautiously. While reduced alpha-diversity in untreated PCOS is generally considered a feature of dysbiosis, reductions in diversity following GLP-1 receptor agonist therapy may instead reflect selective restructuring of the microbial community and enrichment of metabolically favourable taxa (Yin *et al.* 2026). This distinction highlights that microbial composition and functional activity may be more clinically meaningful than diversity indices alone. These agents may influence the microbiome both indirectly through improved metabolic function and potentially through direct effects on microbial composition (Xiong *et al.* 2024). Recent data demonstrate that high-dose GLP-1-based agonists, potentially used in combination with oestrogen, have superior efficacy compared with metformin for managing the metabolic complications of PCOS (Sánchez-Garrido *et al.* 2024). GLP-1 agonists have been reported to decrease inflammatory adipokines, assisting with the restoration of ovulation (Zhang *et al.* 2024). Weight loss reduces visceral adiposity, diminishing pro-inflammatory cytokines, such as IL-18 and the CRP inflammatory marker (Zhang *et al.* 2024).

Probiotic supplementation may become a promising adjunct for PCOS management (Sivasankari & Usha 2022, Martinez Guevara *et al.* 2024). A systematic review demonstrated that probiotic supplementation could produce significant improvements in insulin resistance, lipid profiles, and hormonal function (Martinez Guevara *et al.* 2024). Specific probiotic strains may have targeted effects on PCOS symptoms (Sivasankari & Usha 2022). B*ifidobacterium lactis V9* increases SCFA-producing bacteria, including *Akkermansia, Butyricimonas*, and *Faecalibacterium prausnitzii*, leading to improved brain–gut mediator secretion and reduced LH:FSH ratios (Sivasankari & Usha 2022). *Lactobacillus* supplementation can reduce the free androgen index and malondialdehyde levels, while also improving sex hormone-binding globulin and nitric oxide levels (Sivasankari & Usha 2022). Synbiotic therapy combines probiotics with prebiotics and appears to have an enhanced benefit compared with single interventions (Sivasankari & Usha 2022, Martinez Guevara *et al.* 2024). Synbiotic regimes rich in *Lactobacillus acidophilus*, *Lactobacillus casei*, and *Bifidobacterium bifidum* may help decrease insulin resistance (Sivasankari & Usha 2022).

Other agents may also have a role. Anti-androgens such as spironolactone block androgen receptors, preventing immune alterations in animal models, normalising endometrial uterine NK cell and eosinophil populations (Torstensson *et al.* 2024). Whether anti-androgen therapies directly modulate the microbiome in PCOS remains unclear. Spironolactone is widely used for hyperandrogenic symptoms, and preclinical evidence outside the PCOS context suggests that its effects may partly involve the gut microbiota. In a murine myocardial infarction model, spironolactone altered gut microbial composition, and depletion of the gut microbiota reduced its protective effects, suggesting that microbiome-mediated mechanisms may contribute to some actions of spironolactone (Li *et al.* 2024). However, this evidence is indirect, derived from male mice and a cardiovascular disease model, and cannot be compared directly to women with PCOS. Future studies should assess whether anti-androgen therapy modifies the gut or reproductive tract microbiome in PCOS and whether such changes contribute to improvements in inflammatory, metabolic, or reproductive outcomes.

Pioglitazone improves insulin sensitivity and reduces serum IL-6 and TNF-α (Zhang *et al.* 2024). Several supplements have been studied in the management of PCOS, primarily for their metabolic and antioxidant effects, but their impact on immune function and the microbiome is less established. Myo-inositol can help improve insulin sensitivity and reproductive outcomes, but there is no direct evidence that it modulates immune function or the microbiome in PCOS patients (Kalra *et al.* 2016, Unfer *et al.* 2017, Sharon *et al.* 2024). Coenzyme Q10 (CoQ10) is a potent antioxidant with demonstrated anti-inflammatory effects in PCOS patients, such as a reduction in inflammatory markers (TNF-α, IL-6, and hs-CRP) and downregulation of pro-inflammatory gene expression in peripheral blood mononuclear cells (Nie *et al.* 2023). Studies outside the PCOS context suggest that CoQ10 may modulate immune homeostasis by altering T cell subpopulations and enhancing immune cell activation (Mantle *et al.* 2021). N-acetylcysteine is a precursor to glutathione, which can reduce oxidative stress, lower pro-inflammatory cytokines, and protect against endothelial damage (Thakker *et al.* 2015, Mokhtari *et al.* 2017, Liu *et al.* 2023). These actions may indirectly support immune function in patients with PCOS.

### Future directions

Despite increasing interest in the microbiome–immune axis in PCOS, several important knowledge gaps remain. Future research should prioritise longitudinal cohort studies with standardised sampling protocols and careful phenotypic stratification to clarify temporal relationships between microbiome alterations and the endocrine and metabolic features of PCOS. Integration of microbiome sequencing with immunological and metabolomic profiling may help identify biologically relevant pathways and distinguish disease-specific microbial signatures from broader metabolic dysfunction. Greater focus on patient-centred reproductive outcomes – including ovulation, implantation, miscarriage, and live birth – will also be important to determine whether microbiome-targeted interventions translate into meaningful clinical benefit. Further work is also needed to assess whether treatment-related microbiome changes observed with agents such as metformin, GLP-1 receptor agonists, anti-androgens, and probiotic therapies are directly biologically relevant or secondary to improvements in weight and metabolic function. As evidence evolves, an improved understanding of these interactions may help guide a more personalised and targeted approach to managing patients with PCOS.

## Conclusion

The interaction between PCOS, the reproductive microbiome, and the immune system is a complex, multifactorial relationship with significant implications on disease pathogenesis and response to treatment (Zhou *et al.* 2024). Women with PCOS demonstrate consistent alterations in both gastrointestinal and reproductive microbiomes, characterised by a reduction in beneficial bacteria, an increase in potentially pathogenic organisms, and altered microbial metabolite production (Tu *et al.* 2020, Lu *et al.* 2021, Gu *et al.* 2022). These microbiome changes show clear associations with androgen levels and insulin resistance, with different PCOS phenotypes exhibiting distinct microbial patterns (Yang *et al.* 2024). The immunological consequences of microbiome dysbiosis contribute to chronic inflammation and may directly impact reproductive outcomes (Ichiyama *et al.* 2021, Eslami *et al.* 2024, Zhou *et al.* 2024). Some therapeutic interventions show promising results in targeting the microbiome composition and reducing PCOS symptoms (Martinez Guevara *et al.* 2024, Xu *et al.* 2025). The development of personalised approaches based on individual profiles may further enhance treatment efficacy. As our understanding of these interactions evolves, microbiome and immune-based approaches may become increasingly important components of PCOS management strategies.

## Declaration of interest

Conor Harrity is an Associate Editor of *Reproduction & Fertility* and was not involved in the review or editorial process for this paper, on which he is listed as an author. The authors declare that there are no competing interests that could be perceived as prejudicing the impartiality of the research reported.

## Funding

This research received no specific grant from any funding agency in the public, commercial, or not-for-profit sectors.

## Author contribution statement

PK contributed to writing of the original draft, methodology, and data curation. MWO’R and KM contributed to review and editing. CH contributed to review and editing and supervision. All authors read and approved the manuscript.

## Data availability

No new data were created or analysed during this study. Data sharing is not applicable to this article.

## Ethical approval and consent to participate

This study required no ethical approval.
